# Horizontal viewsheds of large herbivores as a function of woodland structure

**DOI:** 10.1002/ece3.10699

**Published:** 2023-11-09

**Authors:** Amy Gresham, John R. Healey, Markus P. Eichhorn, Owain Barton, Andrew R. Smith, Graeme Shannon

**Affiliations:** ^1^ School of Environmental and Natural Sciences Bangor University Bangor UK; ^2^ School of Biological Sciences University of Reading Reading UK; ^3^ School of Biological, Earth and Environmental Sciences University College Cork Cork Ireland; ^4^ Environmental Research Institute Cork Ireland

**Keywords:** LiDAR, risk perception, terrestrial laser scanning, ungulates, viewsheds, wildlife management

## Abstract

There is great potential for the use of terrestrial laser scanning (TLS) to quantify aspects of habitat structure in the study of animal ecology and behaviour. Viewsheds—the area visible from a given position—influence an animal's perception of risk and ability to respond to potential danger. The management and conservation of large herbivores and their habitats can benefit greatly from understanding how vegetation structure shapes viewsheds and influences animal activity patterns and foraging behaviour. This study aimed to identify how woodland understory structure influenced horizontal viewsheds at deer eye height. Mobile TLS was used in August 2020 to quantify horizontal visibility—in the form of Viewshed Coefficients (VC)—and understory leaf area index (LAI) of 71 circular sample plots (15‐m radius) across 10 woodland sites in North Wales (UK) where fallow deer (*Dama dama*) are present. The plots were also surveyed in summer for woody plant size structure, stem density and bramble (*Rubus fruticosus* agg.). Eight plots were re‐scanned twice in winter to compare seasonal VC values and assess scan consistency. Sample plots with higher densities of small stems had significantly reduced VC 1 m from the ground. Other stem size classes, mean percentage bramble cover and understory LAI did not significantly affect VC. There was no difference in VC between summer and winter scans, or between repeated winter scans. The density of small stems influenced viewsheds at deer eye height and may alter behavioural responses to perceived risk. This study demonstrates how TLS technology can be applied to address questions in large herbivore ecology and conservation.

## Graphical abstract

Viewsheds (the area visible from a given location) can affect animal responses to danger. This study used mobile terrestrial laser scanning (TLS) to reveal that higher densities of small tree stems reduce viewsheds for deer in woodland environments. We discuss potential implications for deer management and demonstrate how mobile TLS can be applied to study the influence of habitat structure on animal behaviour and ecology.

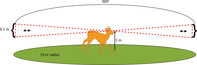

## 1 INTRODUCTION

Remote sensing methods have extensive applications in wildlife ecology research (Kays et al., 2015; Neumann et al., 2015). For example, trail cameras have revolutionized our understanding of animal habitat use and activity patterns at the population level (Green et al., 2020), while GPS tracking has given insight into the processes underpinning complex movement decisions of individual animals (Hebblewhite & Haydon, 2010). Over the past decade, Light Detection and Ranging (LiDAR) methods such as airborne laser scanning (ALS) have been increasingly used to assess how physical habitat structure influences animal ecology and behaviour across a range of taxa in terrestrial and aquatic environments (Acebes et al., 2021; Davies & Asner, 2014; Goetz et al., 2014; Rauchenstein et al., 2022; Wedding et al., 2019). However, when measuring structural characteristics of more closed habitats such as forest understory vegetation, the density and height of the overstory can limit the accuracy of ALS (Campbell et al., 2018; Hull & Shipley, 2019).

Recent reviews have highlighted opportunities for the application of terrestrial laser scanning (TLS) to study habitat structure at a fine scale in forest environments (Aben et al., 2018; Olsoy et al., 2015). For example, studies using static TLS scanners have shown reduced understory vegetation density in forests with high‐density deer populations (Eichhorn et al., 2017; Li et al., 2022), which can lead to degraded habitat quality for birds, particularly woodland specialists (Allombert et al., 2005; Chollet & Martin, 2013; Gill & Fuller, 2007) and small mammals (Buesching et al., 2011; Flowerdew & Ellwood, 2001). Mobile TLS methods differ from static TLS in that the surveyor carries the scanning device and moves through the survey area, which often requires only a single survey as opposed to multiple static surveys. Mobile terrestrial laser scanners may have higher error rates compared to static terrestrial scanners, as the walking speed and pattern of the surveyor influences scan quality (Ryding et al., 2015). However, mobile scanners sample surfaces from multiple angles, which reduces occlusion (Wei et al., 2020) and survey time (Ryding, 2016). With recent technological advances and greater affordability, mobile laser scanners are now capable of providing detailed habitat structure data for the study of animal behaviour (Malhi et al., 2018).

Viewsheds (the area visible from a given location) are affected by the physical structure and density of features such as vegetation and topography (Kuijper et al., 2014; Ndaimani et al., 2013; Parsons et al., 2021), which can influence factors such as predation risk or hunting success (Bellamy et al., 2018; Brown, 1988). In a ‘landscape of fear’ (Gaynor et al., 2019; Laundré et al., 2001; Palmer et al., 2022), behavioural responses to risk induce trade‐offs between concealment, thermoregulation, vigilance and foraging efficiency (Acebes et al., 2013; Glass et al., 2021; Panzacchi et al., 2010; Ratikainen et al., 2007; Wiemers et al., 2014). In dense forest habitats, viewsheds are often restricted to short distances, therefore animal behavioural responses can be shaped by fine‐scale habitat characteristics (Zong et al., 2022). For example, fallen trees and other structural impediments have been shown to reduce ungulate visitation and browsing of vegetation (Hall Defrees et al., 2021; Milne‐Rostkowska et al., 2020; Smit et al., 2012; van Ginkel et al., 2021), possibly due to physical barriers impeding escape routes and detection of predators in forest environments (Kuijper et al., 2013).

In addition to risk from natural predators, perceived risk from human recreational activity (Hagen et al., 2017; van Beeck Calkoen et al., 2022; Wisdom et al., 2018), hunting (Lone et al., 2015; Pecorella et al., 2016), and roads (Eldegard et al., 2012; Karen Marie et al., 2018; Montgomery et al., 2012) influences animal space use and vigilance. This perceived risk is likely to vary with visibility in the environment (Mols et al., 2022; Parsons et al., 2021). For instance, a study of red deer (*Cervus elaphus*) stress responses in Lyme Park, United Kingdom, found that woodland and scrub landscape features decreased the probability of human–deer encounters, which could help buffer stress associated with high human activity (Dixon et al., 2021). Furthermore, a recent study used TLS to assess viewsheds at multiple heights in the vegetation canopy in forest, shrub‐steppe, prairie and desert habitats, and showed that the density, variability and distribution of vegetation is influential for viewshed occlusion (Stein et al., 2022).

There is great potential for TLS studies to quantify viewsheds in forest environments and further our understanding of how physical habitat structure may influence fine‐scale animal space use, foraging behaviour and predation risk (Aben et al., 2018; Lecigne et al., 2020). This has been previously studied at the landscape scale using ALS technology (Parsons et al., 2021). An ALS study found that grizzly bears (*Ursus arctos horribilis*) were less likely to select habitats more visible from roads when resting—indicating selection for safety—but selected more visible areas when travelling—indicating selection for easier passage (Parsons et al., 2021). Another ALS study found that predation risk from human hunters on roe deer (*Capreolus capreolus*) decreased with greater understory density, probably due to reduced sightline length impeding shooting accuracy, while predation risk from an ambush predator, the Eurasian lynx (*Lynx lynx*), increased (Lone et al., 2014). Most recently, a study in the Bavarian Forest National Park, Germany, combined ALS and static TLS to study how visibility influenced movement rates of red deer in relation to risk perception (Zong et al., 2022).

Our study aimed to evaluate the extent to which woodland structure influences horizontal visibility at a height relevant to a large herbivore species ‐ fallow deer (*Dama dama*). We used mobile TLS to quantify horizontal viewsheds, summarised as Viewshed Coefficients (VC) 1 m above the ground. Woodland structure was assessed by surveying the density of different stem size classes, species composition of woody vegetation (trees and shrubs) and bramble (*Rubus fruticosus agg*.) cover. The expectation was that higher densities of tree stems of all size classes and higher bramble cover would significantly reduce horizontal visibility as a function of distance from a given point. Leaf area index (LAI) of the understory was also extracted from the TLS data to assess the extent to which leafy foliage influenced horizontal viewsheds. We predicted that higher LAI values would correspond to lower horizontal visibility as a function of distance from a given point. In addition, a subset of plots was scanned in both summer and winter to compare horizontal viewsheds in different seasons. Visibility may be reduced in leaf‐on compared with leaf‐off conditions due to heightened seasonal foliage density from deciduous vegetation. Each winter scan was also repeated to check the consistency of the mobile scanning method. Through this work, we demonstrate how potential sightlines of large herbivores are altered by properties of forest understory structure.

## 2 METHODS

### 2.1 Study area

**FIGURE 1:**
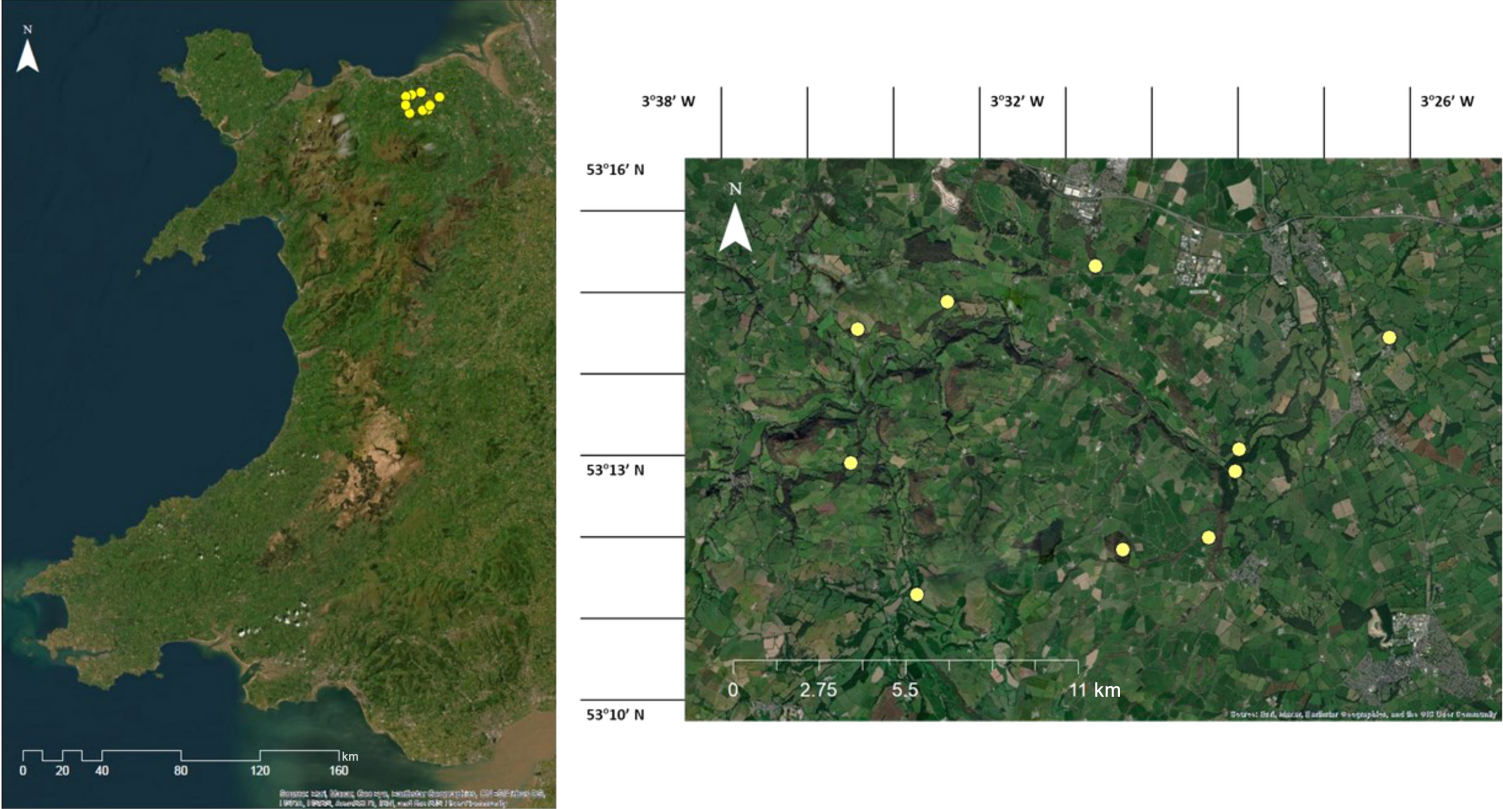
Maps of the Elwy Valley study region in North Wales, United Kingdom. The yellow dots show the position of the 10 woodlands containing the 71 circular sampling plots surveyed for this study. Maps generated using ArcGIS Desktop © 1999–2020, Sources: Esri, DigitalGlobe, GeoEye, i‐cubed, USDA FSA, USGS, AEX, GetMapping, Aerogrid, IGN, IGP, swisstopo and the GIS User Community.

**FIGURE 2:**
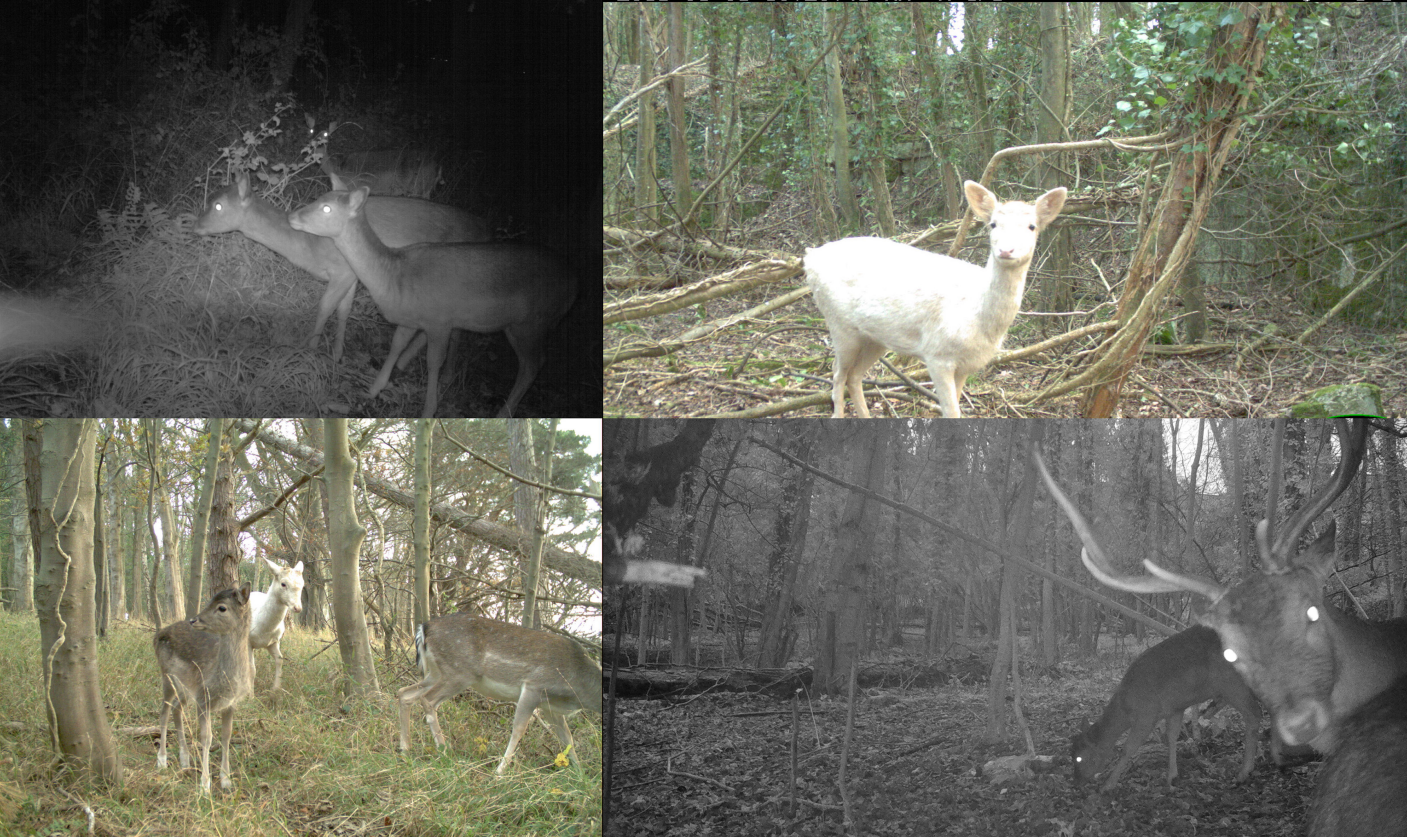
Photographs of fallow deer (*Dama dama*) from trail cameras deployed by O Barton in the Elwy Valley study area. No other deer species were captured on the trail cameras for the duration of the study.

Ten woodland study sites were established in the Elwy Valley, North Wales (Figure 1). The Elwy Valley is a landscape mosaic of farmland (predominantly livestock pasture and forage crops) and patches of woodland under different ownership and management objectives. These woodlands vary in composition and maturity, and included conifer plantations, mixed broadleaf‐conifer woodland and semi‐natural broadleaf woodlands (see Appendix S1 for details on species composition of each site). There is a population of approximately 1500 fallow deer occupying this area (Figure 2; Lee Oliver, personal communication, Game & Wildlife Conservation Trust).

**FIGURE 3:**
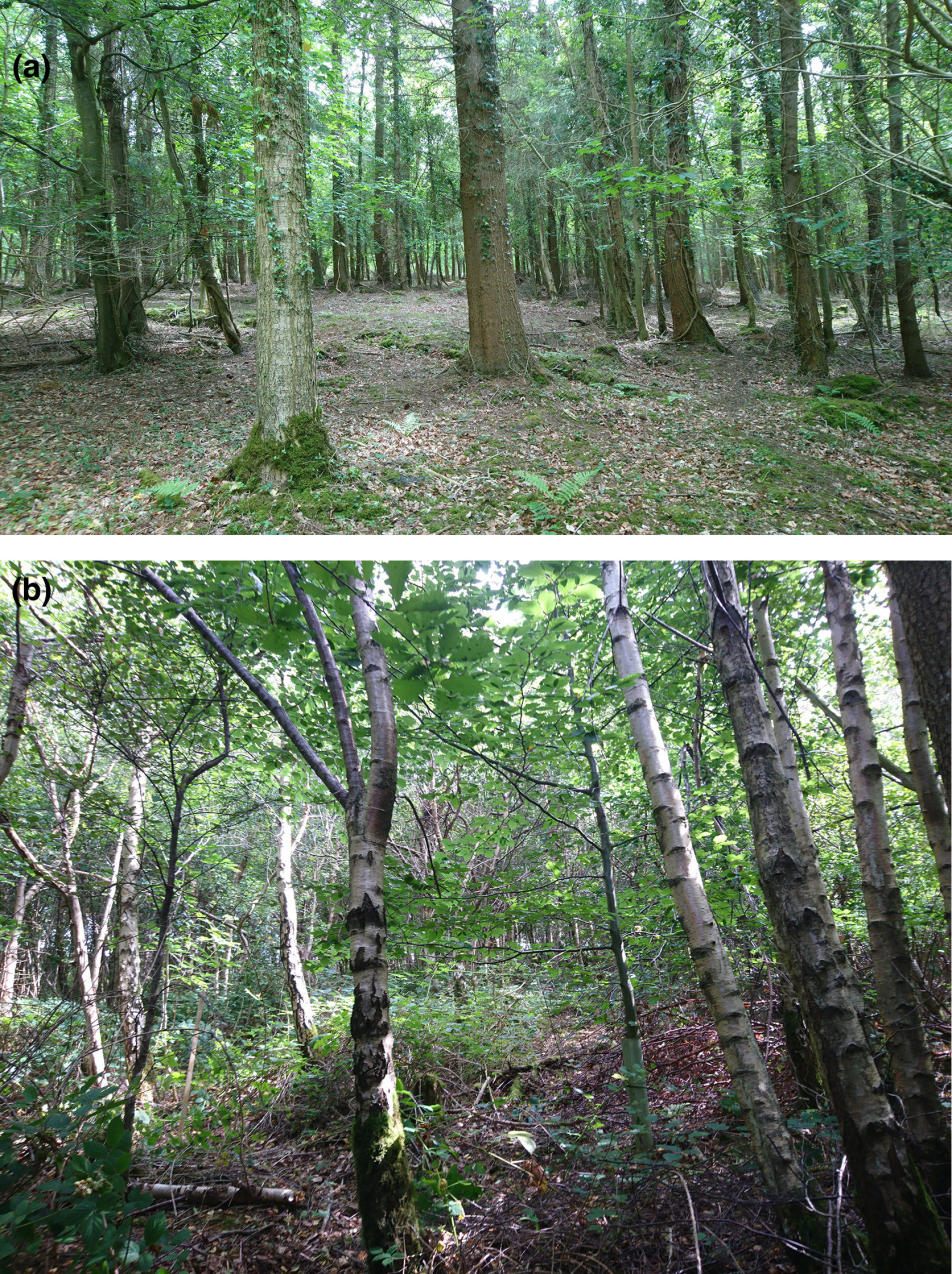
(a) Site HFD in summer 2019. There is a notable browse line from deer herbivory and lack of dense understory vegetation, with most trees belonging to larger size classes. (b) Site EWD in summer 2020. The understory is relatively dense with many smaller trees and dense bramble cover.

Circular plots (15‐m radius) were located to capture as much variation as possible in density, structure, size and diversity of the tree and shrub communities within each of the 10 woodland sites (Figure 3). Table 1 shows the number of individual study plots per site. Sample plots were positioned to avoid human‐constructed paths or roads, although these features were sometimes close to plot edges. Sites WFR, TCL and MRN had some very steep slopes which could not be surveyed due to safety constraints. Woodland edges were not avoided.

**TABLE 1:** Sample size and area of the 10 woodland study sites.

Study site	Number of study plots	Site area (ha)
BLH	6	5
BWN	8	11
EWD	6	12
EWW	10	20
HFD	10	64
LNH	8	10
MRN	5	6
PCG	7	12
TCL	4	2
WFR	7	11

### 2.2 Data collection

LiDAR scans were conducted with a GeoSLAM (Nottingham, UK) ZEB Revo TLS system to determine the horizontal visibility and LAI in each plot. Previous studies have validated GeoSLAM ZEB devices for use in forest surveys (Bauwens et al., 2016; Camarretta et al., 2021; Ryding, 2016). This device had a relative accuracy of 1–3 cm. Each of the 71 plots was scanned once in August 2020. The conditions required for these surveys were no rain and wind speeds of <16 km/h. This reduced the risk that rain or moving foliage would artificially elevate point density. The GeoSLAM device was placed on the ground at the centre of the plot during set‐up to mark the start and finish point. The scanning procedure involved the same surveyor walking around and through each 15‐m radius circular plot multiple times for 15–20 min, with the scanner held at breast height. During the scan, care was taken to present the scanner to habitat features from several angles to minimize occlusion effects. The walking pattern consisted of walking to the edge of the plot, walking around the edge in both directions, then crossing the plot from different angles in a closed loop, starting and finishing in the plot centre (Bauwens et al., 2016; Ryding, 2016). Areas with thick cover of shrubs or scrambling plants, for example, bramble and blackthorn (*Prunus spinosa*), were surveyed as thoroughly as possible.

Scans were also conducted in a subset of eight plots in winter (January 2021) to compare horizontal visibility in leaf‐off versus leaf‐on seasons. This January sampling period was also used to assess the consistency of the scanner and the data collection methodology by repeating all scans in the eight sampling plots, one directly after the other. The two scans per plot were then compared for significant differences in horizontal visibility.

All trees, saplings and shrubs (hereafter referred to as ‘woody plants’) greater than 0.3 m in height were surveyed in each plot. For each woody plant, the taxon was identified as precisely as possible (usually species, otherwise genus). For woody stems taller than or equal to breast height (1.3 m), the size class of diameter at breast height (DBH) was determined using a diameter tape (see Table 2 for details of size class classification). For multi‐stemmed woody plants, the DBH of the largest stem was measured and the total number of stems was counted. For saplings shorter than breast height, the height was measured using a metre ruler. Woody plants less than 0.3 m in height were not recorded. Both dead and living woody plants were included in the inventory. In two plots at site LNH, there was very dense growth of saplings and small trees, particularly ash (*Fraxinus excelsior*). To enable measurement of these saplings within a practical timeframe, all ash stems within the ‘Sapling’ and ‘Small’ categories (Table 2) within plot LNH4 were counted in a circular sub‐plot (4.5‐m radius) at the plot centre, then these counts were scaled up to estimate the number of ash saplings in the 15‐m radius plot area. The same approach was used for ‘Saplings’ and ‘Small’ stems of all tree species in plot LNH8.

**TABLE 2:** Woody plant stem size class categories from the woodland surveys.

Category name	Woody plant size category
Sapling	>0.3 m, <1.3‐m height
Small	≥1.3 m height, <10‐cm DBH
Medium	10–20 cm DBH
Large	21–30 cm DBH
Very large	≥31 cm DBH

Each plot was surveyed for bramble cover either two or three times across the summers of 2019–2021 using 0.25‐m^2^ quadrats sub‐divided into 25 × 0.01 m^2^ squares. For each survey, eight quadrats were randomly placed inside the plot using cardinal directions and distance from the plot centre (1–15 m). At each of these eight locations, a quadrat was placed on the ground and the number of squares containing bramble foliage and stems was counted from above. These eight counts were averaged to obtain a bramble count value for each plot survey. These two or three values from across the survey years were then averaged to obtain mean percentage bramble cover for each plot.

### 2.3 Data analysis

Point clouds were processed in R version 4.0.3 (R Core Team, 2021) using the *viewshed3d* (Lecigne et al., 2020; Lecigne & Eitel, 2022) and *lidR* (Roussel et al., 2020) packages. Due to the memory constraints of a standard computer, the analysis was run on the Supercomputing Wales platform. The processing broadly followed example workflows in the *viewshed3d* handbook. Each cloud was first cropped to a 15‐m radius using the *sample_scene* function from the *viewshed3d* package. Duplicate points were removed using the *filter_duplicates* function from the *lidR* package, then isolated points were removed using the *denoise_scene* function (*viewshed3d*). The ground points were classified using the *classify_ground* function (*lidR*). The topographical slope was removed using the *remove_slope* function (*viewshed3d*) to make sure that the effect of vegetation in each plot could be examined independently of slope. Finally, the ground was reconstructed with the optimal resolution to ensure that sightlines did not pass through the forest floor using the *reconstruct_ground* function (*viewshed3d*).

**FIGURE 4:**
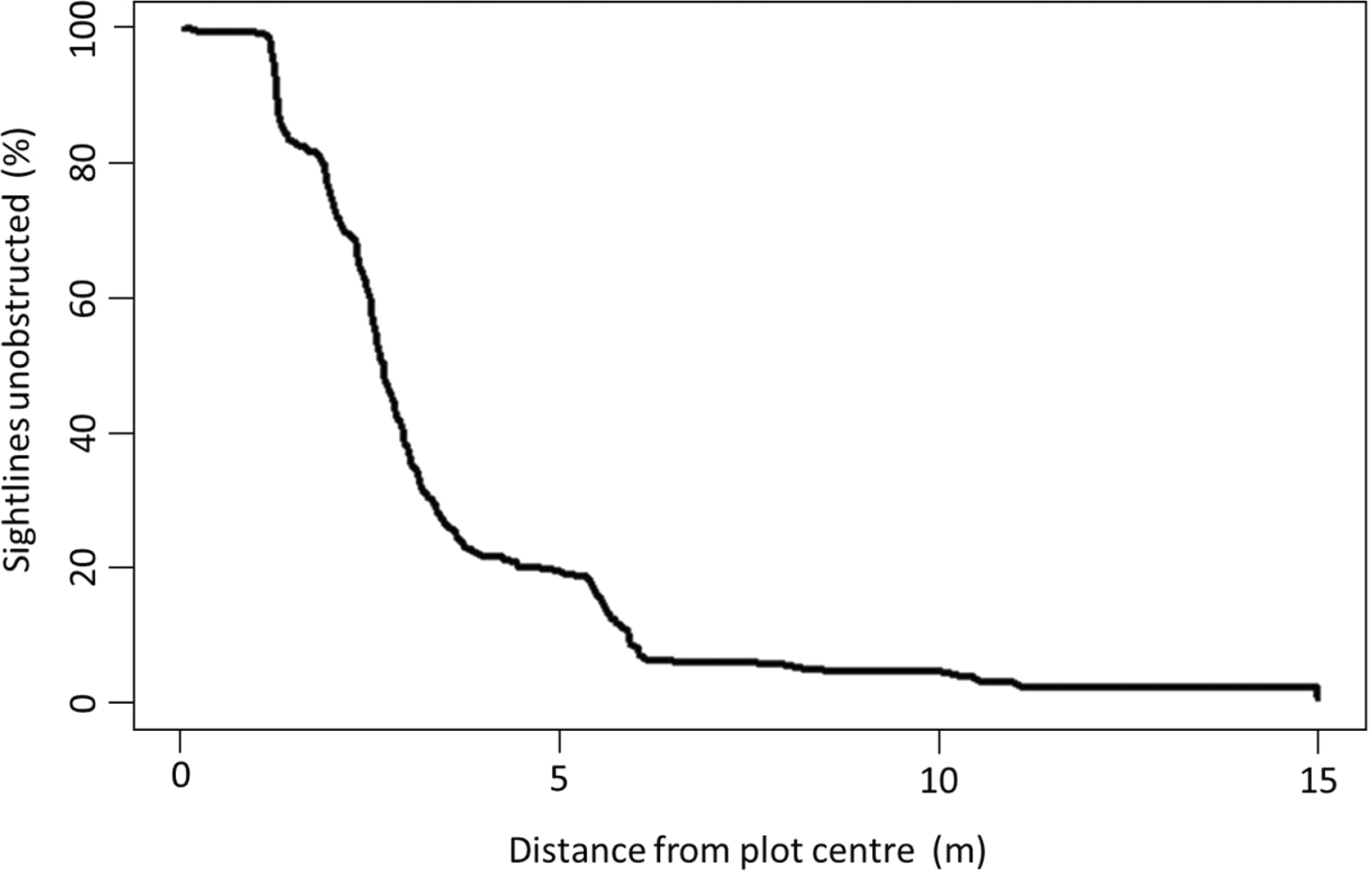
An example curve of percentage horizontal visibility 1 m above the ground surface. Percentage horizontal visibility (unobscured sightlines) declines with distance from 0 m (plot centre) as objects obstruct the view. In this example, the visibility declines sharply between 1 and 3 m from the plot centre. The Viewshed Coefficient (VC) represents the total area under the curve of percentage visibility for each circular sampling plot.

The VC was calculated using the *h_visibility* function within the *viewshed3D* package. The VC is defined as ‘the area under the curve of visibility as a function of distance from the animal's location’ (Figure 4) (Lecigne & Eitel, 2022).

**FIGURE 5:**
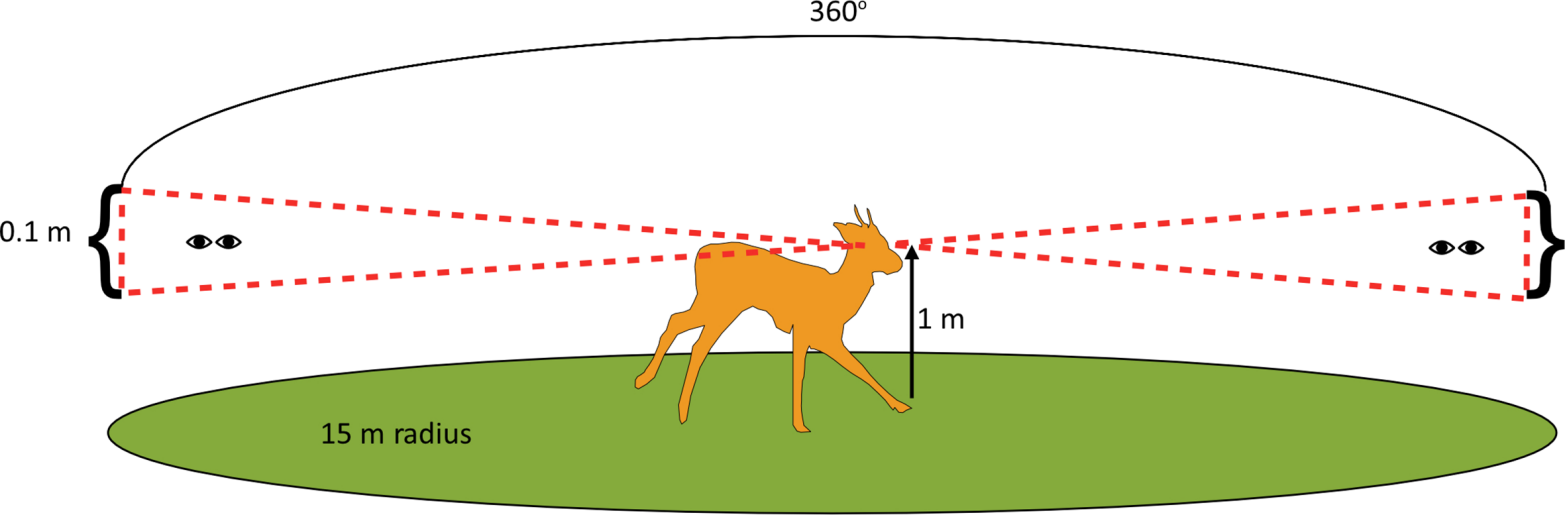
Illustration of the viewshed concept in a 15‐m radius circular sampling plot in a study woodland. The dotted red lines show the shape of the biconcave disc within which the viewsheds are measured. The Viewshed Coefficient (VC) calculation assumes the deer is at the centre of the plot with a horizontal sightline 1 m above the ground surface. The VC encompasses a 360‐degree view at this height with an angular resolution of one degree and a maximum viewshed thickness of 0.1 m.

The location of the deer in each plot was defined using XYZ coordinates 0, 0, 1. This placed the animal at the centre of each plot and 1 m above the ground surface. Fully grown fallow deer females stand at 0.7–0.8 m at the shoulder, while fully grown males stand at 0.7–0.9 m (Putman, 1989). Therefore, the VC was a representation of visibility at the eye height of fallow deer standing in the centre of the plot over a 360 degree viewshed as a biconcave disc with a maximum thickness of 0.1, 1 m from the ground (Figure 5).

Leaf Area Index (LAI) values were calculated for each point cloud within the bounds of 0.75–1.5 m in height. Point cloud processing used the same functions as for the Visibility Coefficient estimates, except for the *reconstruct_ground* function. In addition, the *filter_poi* and *clip_poi* functions (*lidR*) were used to crop the point cloud to 2 m in height and 15 m in radius, respectively. The data were then filtered to include the z coordinates only, then a leaf area density (LAD) profile was generated for each point cloud at height bands of 0.75, 1.25 and 1.75 m using the *LAD* function from the *lidR* package. The LAI for each point cloud was calculated from the LAD profiles for the height range of 0.75–1.5 m using the *lai* function in the *leafR* package (de Almeida et al., 2021).

The large number of woody plant species across the 10 sites (*n* = 44), combined with the high level of variability among plots in species composition, meant that there were no clear relationships between species and VC that could be demonstrated statistically. While certain species provided a notably strong obstruction of view, such as patches of large *Cotoneaster* spp. and cherry laurel (*Prunus laurocerasus*) evergreen shrubs at site BWN, they occupied an insufficient number of plots to test the individual effects of these species. Our analysis therefore focused primarily on the effects of woody plant size, understory LAI and bramble cover on VC. Stem counts within the 15‐m radius plots were converted to stems per hectare for further analysis.

Prior to analysis, collinearity between fixed effects was examined in a correlation matrix. Sapling density and small stem density were found to be significantly correlated (*r* = .74). In addition, data exploration using dot plots, histograms and box plots was conducted for each of the fixed effects and the dependent variable (VC) to check whether a normal error structure was appropriate. Based on this data exploration, a log transformation was applied to correct zero‐skewness in the following variables: very large stem density, small stem density, sapling density and mean percentage bramble cover. In addition, all explanatory variables were scaled through *z*‐scoring to bring them on to comparable scales for analysis. Using the R package *lme4* (Bates et al., 2015), a global linear mixed model including every explanatory variable was then analysed using the *dredge* function from the *MuMIN* package (Bartoń, 2022), with the condition that small tree density and sapling density did not co‐occur in any models due to their strong collinearity.

To gain an understanding of how shade from larger trees may have influenced understory density and resulting viewsheds, we classified the species of all medium, large and very large woody plants (mature stems) by their propensity to cast shade using values reported by Ellenberg (Ellenberg, 1988) (p. 50) (Appendix S2). These values were on a scale of increasing shade from one to six: extremely low, very low, low, medium, high and very high. Where species from the study plots were not included in the original classification table, a category was assigned based on a close relative in the table, or by expert opinion (JR Healey). The average shade value of each survey plot was then calculated. Ellenberg values have previously been used to obtain average estimates for abiotic conditions in forests (Boulanger et al., 2015). A linear mixed model was used to examine the relationship between average Ellenberg value and log small stem density, with site as a random effect.

The following statistical tests were also conducted on the VC values: (1) point cloud processing was repeated without the slope removal step to assess whether the slope of the ground influenced VC. We compared VC values of point clouds from the same plots with and without ground slope removed using a two‐tailed Wilcoxon signed‐rank test, (2) we used a one‐tailed Wilcoxon signed‐rank test to determine whether there was any significant difference between the VC values of plots surveyed in the summer and winter. A mean value of VC from each of the eight pairs of winter scans was taken, and these were then compared with the eight summer scans from the same plots, and (3) we used a two‐tailed Wilcoxon signed‐rank test to compare same‐day repeat winter scans to assess whether error in the methodology generated differences in VC between scans. Scan pairs were randomised into two groups (A, *n* = 8 and B, *n* = 8) prior to this paired test to remove the influence of any order effects.

## 3 RESULTS

### 3.1 Examining the effects of woody stem size structure on horizontal viewsheds

A total of 71 VC values from summer scans of individual sample plots across 10 woodland sites were used in the analysis. Across the 71 plots, 20,555 woody plant stems in 37 genera (Appendix S1) were recorded, which included the amended counts for plots LNH8 and LNH4. Of these counted stems, 601 were dead. Calculated stem densities per size class are shown in Appendix S3.

**FIGURE 6:**
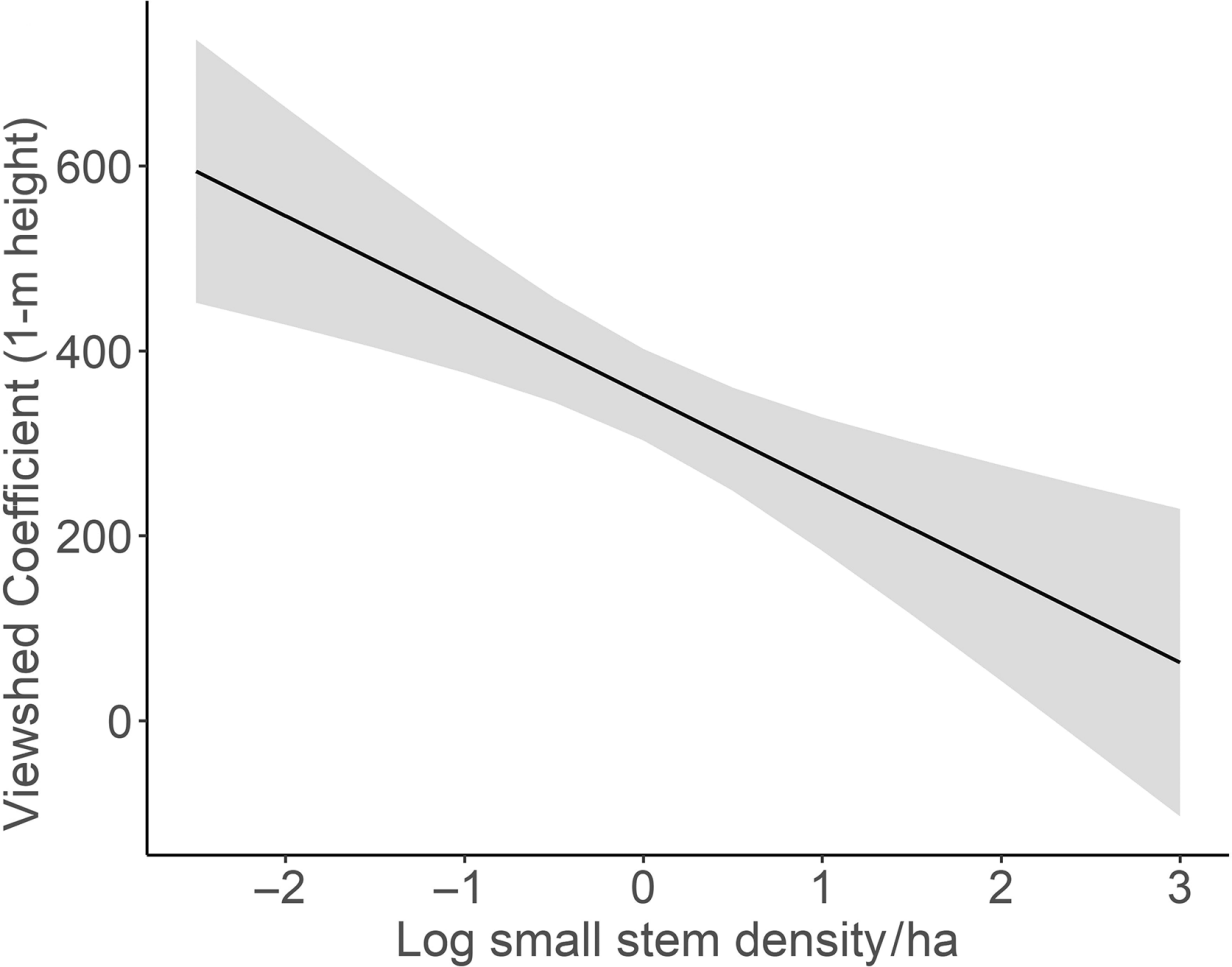
Predicted values from the top model for predicting Viewshed Coefficient (y) as a function of log small stem density (x). The error around the line represents the 95% confidence intervals of the predicted values.

The global model containing small tree density (not sapling density) had a ΔAIC of 0, while the next top ranked model had a ΔAIC of 5.20. In addition, the top model had an AIC weight of 80%. Given the importance of the top model, the estimates and 95% confidence intervals were examined for each explanatory variable. VC significantly decreased with increasing density of small stems (*β* = −103.84, 95% CI = −149.67, −58.00, Figure 6, Table 3). The density of the remaining stem size categories and understory LAI did not have a significant effect on VC (Table 3). Despite there being notable bramble cover in most of the study sites (Appendix S4), average percentage bramble cover did not significantly affect VC (Table 3). A linear model showed that logged density of small stems was negatively related to average strength of shade from mature trees (*β* = −0.45, 95% CI = −0.80, −0.09, Appendix S5).

**TABLE 3:** Model estimates and 95% confidence intervals for each of the variables that featured in the top model (ΔAIC = 0).

Fixed effect	Lower 95% CI	Estimate	Upper 95% CI
**Log small stem density**	**−149.67**	**−103.84**	**−58.00**
Medium stem density	−49.99	−3.57	42.86
Large stem density	−70.04	−27.04	15.97
Log very large stem density	−16.15	33.40	82.95
Log average percentage bramble cover	−70.01	−25.05	19.91
Leaf area index (0.75–1.5 m)	−38.04	1.93	41.90

Bold values indicate a significant effect as the confidence intervals do not overlap zero.

### 3.2 Topographical slope

Mean VC was marginally higher when the ground slope was removed (mean = 347.80, SD = 199.87, *n* = 71) than when the ground slope was included (mean = 334.36, SD = 203.48, *n* = 71) during point cloud processing. However, the difference was not significant (mean difference = −13.44, SD = 78.07) between point clouds with and without slope included (V = 1184, *p* = .59).

### 3.3 Season

**FIGURE 7:**
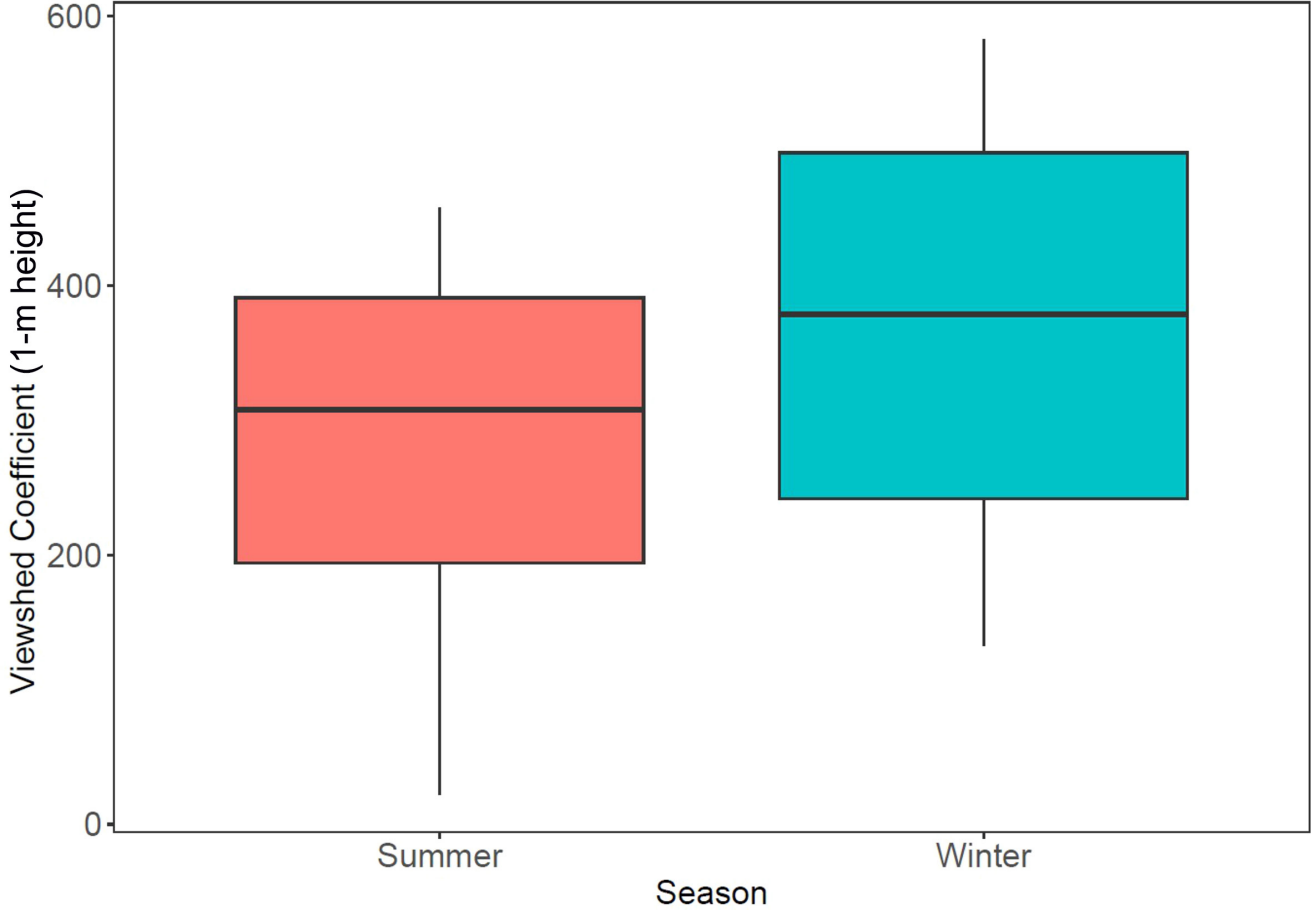
Viewshed Coefficients from eight study plots scanned in summer (orange box) and again in winter (blue box). Ground slope was removed. The central black lines show the median, the boxes show the upper (75%) and lower (25%) quartiles and the tails show the minimum and maximum values.

Mean VC was higher in winter scans (mean = 366.87, SD = 168.24, *n* = 8) than in summer scans (mean = 280.91, SD = 148.51, *n* = 8), but the difference was not significant (mean difference = −85.96, SD = 89.22) between the VCs of winter and summer scans (V = 11, df = 7, *p* = .84, Figure 7).

### 3.4 Scan consistency

The mean VC for winter scans in group A (mean = 367.78, SD = 158.93, *n* = 8) and group B (mean = 365.96, SD = 181.17, *n* = 8) were very similar. There was no significant mean difference (mean difference = −1.82, SD = 54.33) between the VCs of scans in groups A and B (V = 16, df = 7, *p* = .84). This indicates that the walking pattern of the surveyor did not influence the outcome of the viewshed analysis.

### 3.5 Power analysis

Given the small sample size used in the above Wilcoxon signed‐rank tests (*n* = 8), we conducted a post hoc power analysis to gauge the effect size that would be required to generate a significant effect. This was conducted using the *pwr.t.test* function from the *pwr* package (Champely, 2020). With a minimum power of 0.8, a sample size of 8 and a significance level of .05, the effect size required would be 0.98. Therefore, the probability of a type 1 error was probably very high when performing these tests.

## 4 DISCUSSION

Woodland plots with a high‐density of small woody stems had lower horizontal visibility. Small stems occurred at high density compared to other size categories (Appendix S3). This fits with the gap‐phase paradigm in forest ecology: openings in the canopy due to windthrow or disease allow light to reach the forest floor, which stimulates seed germination and growth of previously shaded seedlings, resulting in patches of high density small woody stems (Attiwill, 1994). This was evident for the pioneer species birch (*Betula* spp.) and light‐demanding species ash (*Fraxinus excelsior*) at several study sites (Appendix S1). In addition, hazel coppice probably contributed to reduced VC, particularly at site EWW (Appendix S1).

The density of larger stem size classes (medium, large and very large) had negligible independent effects on VC. The density of larger tree stems is restricted by their greater resource requirements. In addition, the foliage of larger trees is generally concentrated in the main canopy, above the eye height of terrestrial herbivores. Therefore, they are less likely to significantly hinder viewsheds at 1 m. Canopy trees can influence the understory through shading from dense foliage, which reduces the density of light‐demanding understory vegetation (Coomes et al., 2005; Ellenberg, 1988). In our study sites, this was especially true of plots that contained beech (*Fagus sylvatica*) or hornbeam (*Carpinus betulus*), which cast especially heavy shade (Ellenberg, 1988). This is supported by our examination of the density of small stems using Ellenberg's species shade values (Ellenberg, 1988), which indicated that plots with a canopy dominated by trees casting a heavier shade had lower densities of small stems.

Mean percentage bramble cover had no significant effect on VC. Bramble cover can become depleted in woodlands with heavy deer browsing (Cooke & Farrell, 2001; Gill & Fuller, 2007), but was nonetheless prevalent across most of our study plots and was particularly dominant at sites EWD and TCL (Appendix S4). The lack of an effect may be because bramble cover was concentrated in the field layer, which was rarely above 1 m in height (Appendix S6). At several sites, the fallow deer were using bramble patches as refugia, with deer‐sized hollows inside some of the thickets and lots of deer faecal droppings in the vicinity (A. Gresham, personal observation). While we did not find a significant effect of bramble cover on VC at 1‐m height, it may be that localised thickets serve as an important component of habitat structure for animals seeking cover.

Understory LAI was not a significant predictor of VC in the summer scans. This may be because there was very little variation in understory LAI (Appendix S7). This could be symptomatic of widespread browsing by the abundant deer population reducing structural complexity of the understory (Eichhorn et al., 2017) or dense canopy foliage restricting light availability to lower layers. Both mechanisms could lead to the low density of saplings relative to larger stems found in the woody plant surveys at most sites (Appendix S3). Sapling stem density did not feature in the top model, supporting the notion that saplings and associated foliage have very little influence on horizontal visibility, particularly given their sparse occurrence across the study plots.

The lack of variation in LAI may also be due to the limitations of the data collection methods using TLS (Wang & Fang, 2020) and/or the methodology used to generate the LAI values. While LAI has typically been used at a coarse resolution to evaluate ecosystem processes and environmental conditions, advances in TLS technology have led to LAI being measured at a similar spatial scale and resolution to this study, examining individual forest stands (Wei et al., 2020; Zheng et al., 2013; Zhu et al., 2020). LAI is a two‐dimensional measure of the per unit projection leaf area on the ground calculated from a canopy height profile of LAD, which is a three‐dimensional measure of leaf area per unit volume (Wei et al., 2020). In our study, LAI was estimated based on a LAD height profile of 0.75–1.5 m, while the response variable (VC) was measured in a narrow band at 1‐m height. Therefore, LAI may not have been the best measurement for estimating how foliage affected visibility at such a specific height. Calculating LAD for the specific 1‐m height band may have provided a better measure of how foliage influenced visibility. We suggest that future studies using LiDAR to investigate how understory foliage influences habitat structure and visibility employ 3D foliage density metrics rather than 2D measures such as LAI.

Horizontal visibility was greater in the winter scans than in the summer scans of the same plots, but the difference was not significant. Although the direction of the effect was as expected, this finding goes against our expectation that visibility would be much greater in winter due to loss of deciduous leaves. The lack of seasonal difference may be linked to the minimal variation summer foliage density within the understory, indicated by the LAI data (Appendix S7) and the overall low density of saplings across the sites. The repeated winter scans showed that the scanning methodology produced consistent VC values, indicating that this technology is a reliable method for measuring and comparing horizontal viewsheds. However, our interpretation of these results is limited by a low sample size as indicated by the power analysis, with just eight plots used for the seasonal comparison and eight repeated scans for the consistency test.

Exclusion of topographical slope during point cloud processing did not significantly alter VC. This does not, however, confirm whether slope is an important factor for deer refuge in the study area. Topographical slope has been shown to affect viewsheds and ungulate browsing behaviour at the landscape scale using digital elevation models (DEMs) (Ndaimani et al., 2013; Roženbergar et al., 2019). When exposed to increased disturbance, ungulates may select for more rugged terrain where there is reduced hunter access and increased vegetation cover (Buchanan et al., 2014; Sergeyev et al., 2020). In landscapes like the Elwy Valley with steep topography and frequent human disturbance from culling and recreation, it would be interesting to examine the effects of slope on viewsheds at a landscape scale, but this is outside the scope of this study.

Hunting takes place in the Elwy Valley, for both recreation and management of the fallow deer population. It is a good practice for hunters to ensure a clear line of sight before making a shot; this reduces the risk of deer being disturbed and escaping the cull or an unclean shot leading to wounding and distress of the animal (Aebischer et al., 2014). Therefore, where humans are the only predator and adopt a ‘sit and wait’ shooting strategy—the main method of hunting in the study area—open areas are likely to present the greatest risk to the deer (Lone et al., 2015; Meisingset et al., 2022; Norum et al., 2015). For example, a study on the Swedish–Norwegian border found that the probability of moose (*Alces alces*) being killed by human hunters increased with reduced terrain ruggedness and greater distance to bogs and young forests, indicating that hunters mostly killed moose in more easily accessible, open areas (Ausilio et al., 2022). In the present study, plots with higher densities of small stems had shorter average viewsheds, which may reduce both the perceived and actual threat from human hunters compared with plots that had lower densities of small stems.

This study has demonstrated a novel application of mobile TLS for studying the effects of fine‐scale habitat structure on large herbivore behaviour and ecology. There are numerous possible applications of the rapid quantification of habitat structure that mobile TLS provides, such as the study of viewsheds for multiple animals at different vantage points in the same system (Lecigne et al., 2020; Lecigne & Eitel, 2022) or across different ecosystems (Stein et al., 2022). For example, Lecigne et al. (2020) used TLS data to compare how forest structure influenced the viewsheds for an airborne predator, a terrestrial predator and a shared terrestrial prey species, which may affect the success of predation attempts.

It is important to consider that individuals of the same species differ in size and behaviour, therefore visibility measures at a set height may not apply to all individuals. In cervids such as roe deer, young offspring may have a lower field of view than their adult counterparts, especially as they rely on bedding down as their main anti‐predation strategy in the first few weeks of life, as opposed to standing and fleeing (Christen et al., 2018; Jarnemo, 2002). In addition to different demographic groups, vantage points can change for the same individual depending on its activity state. As ruminants with a digestive system relying on pre‐gastric fermentation, cervids spend significant periods in a reclined position with a lower vantage point compared to a standing position, which could both conceal them from predators and reduce their ability to perceive danger. A recent study combining ALS and static TLS accounted for this by quantifying red deer habitat selection in relation to visibility using averaged three‐dimensional cumulative viewsheds for eye lines of bedded deer (30 cm) or standing deer (140 cm) (Zong et al., 2022). In addition, other metrics such as foliage density could be used to study the shelter quality of vegetation for thermoregulation or seasonal forage availability (Hill & Broughton, 2009; Li et al., 2018). For example, a roe deer study used ALS to quantify how canopy and understory cover influenced habitat selection according to wind speed and snow depth (Ewald et al., 2014). These concepts may be of interest for future research using TLS to address behavioural trade‐offs relating to fine‐scale habitat structure in animal populations (Davies & Asner, 2014; Olsoy et al., 2015; Vierling et al., 2008).

## 5 CONCLUSIONS

We used a novel 3D mobile TLS approach to demonstrate that higher densities of small woody stems reduced horizontal visibility at 1‐m height above the ground, while foliage quantities as measured by LAI and average bramble cover had no significant effect. Higher densities of small stems occurred in plots with less shade from canopy trees. High densities of small woody stems may break up sightlines in the understory and reduce perceived and/or actual threat levels for large herbivores—particularly the risk associated with human hunters. Behavioural responses to perceived risk may be related to understory structure in temperate forests. The study of viewsheds using terrestrial LiDAR has great potential for improving our understanding of how habitat structure influences animal behaviour.

## AUTHOR CONTRIBUTIONS

**Amy Gresham:** Conceptualization (lead); data curation (lead); formal analysis (equal); funding acquisition (supporting); investigation (lead); methodology (lead); project administration (equal); resources (lead); software (equal); validation (lead); visualization (lead); writing – original draft (lead); writing – review and editing (equal). **John R. Healey:** Conceptualization (supporting); formal analysis (supporting); funding acquisition (lead); methodology (supporting); resources (supporting); supervision (supporting); writing – review and editing (equal). **Markus P. Eichhorn:** Conceptualization (supporting); formal analysis (supporting); funding acquisition (lead); methodology (supporting); supervision (supporting); writing – review and editing (equal). **Owain Barton:** Methodology (supporting); visualization (supporting); writing – review and editing (supporting). **Andrew R. Smith:** Formal analysis (supporting); methodology (supporting); writing – review and editing (supporting). **Graeme Shannon:** Conceptualization (supporting); formal analysis (equal); funding acquisition (lead); methodology (supporting); project administration (equal); resources (supporting); software (equal); supervision (lead); visualization (supporting); writing – original draft (supporting); writing – review and editing (equal).

## CONFLICT OF INTEREST STATEMENT

The authors declare no conflict of interest.

## Supporting information


Appendices S1–S7.

## Data Availability

The data that support the findings of this study are available in the following Dryad repository: https://doi.org/10.5061/dryad.zcrjdfnjm.
