# Supplementary material for: Horizontal viewsheds of large herbivores as a function of woodland structure
Source: Ecol Evol. 2023 Nov 9;13(11):e10699. doi: 10.1002/ece3.10699 (PMC10636313; doi:10.1002/ece3.10699)
Supplement: Supplementary file 1 — Appendices S1–S7. [file ECE3-13-e10699-s001.docx]

## Appendix

**S1:** Percentage of woody plant stems belonging to different genera across the ten sampling sites, split by stem size. Each bar plot contains data for a different stem size category, and the columns represent data from the ten sites. Dominant genera include ash (Fraxinus), birch (Betula), hazel (Corylus) and oak (Quercus).


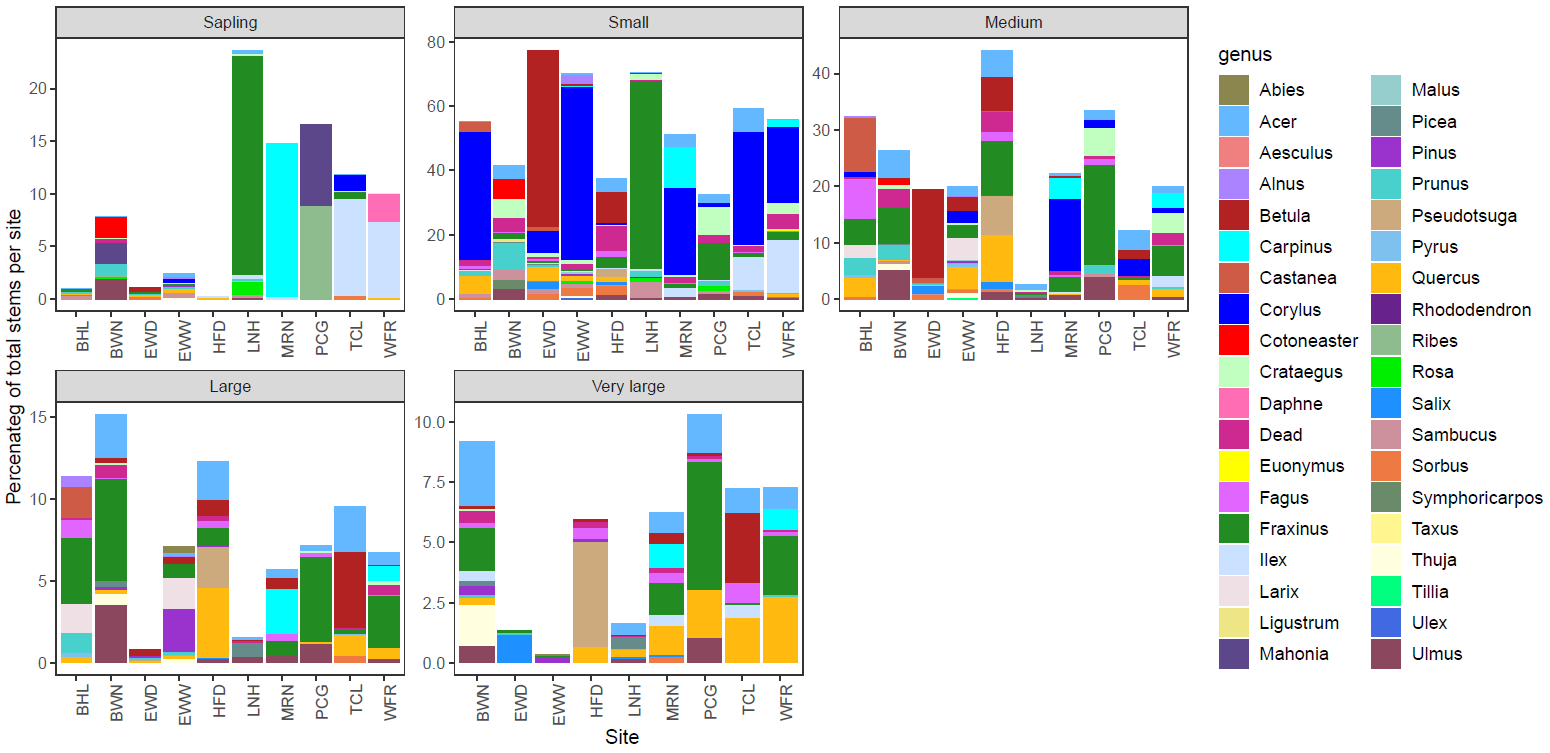


**S2:** Woody plant taxa identified in the medium, large or very large stem diameter categories with their corresponding shade-casting categories (Ellenberg, 1988) (p. 50).

| Taxon | Shade casting category |
| --- | --- |
| *Abies* spp | High |
| *Acer campestre* | Medium |
| *Acer pseudoplatanus* | High |
| *Acer* spp | Medium |
| *Alnus rubra* | Medium |
| *Betula* spp | Very low |
| *Carpinus betulus* | Very high |
| *Castanea sativa* | Medium |
| *Corylus avellana* | Medium |
| *Cotoneaster* sp | Very high |
| *Crataegus* spp | Low |
| *Dead* | Extremely low |
| *Euonymus europaeus* | Low |
| *Fagus sylvatica* | Very high |
| *Fraxinus excelsior* | Medium |
| *Ilex aquifolium* | Very high |
| *Larix decidua* | Very low |
| *Malus spp* | Low |
| *Picea sitchensis* | Very low |
| *Pinus sylvestris* | Very low |
| *Prunus avium* | Medium |
| *Prunus laurocerasus* | Very high |
| *Prunus spinosa* | Low |
| *Pseudotsuga menziesii* | High |
| *Pyrus* spp | Low |
| *Quercus rubra* | Medium |
| *Quercus* spp | Medium |
| *Salix caprea* | Low |
| *Salix* spp | Low |
| *Sambucus nigra* | Low |
| *Sorbus aucuparia* | Low |
| *Thuja occidentalis* | High |
| *Thuja plicata* | High |
| *Tillia cordata* | High |
| *Ulnus glabra* | High |

**S3:** Stem density per hectare per sample plot, split by site and coloured by woody plant size category. Woody plant size categories were defined as follows: Sapling (> 0.3 m, < 1.3 m height); Small (> = 1.3 m height, < 10 cm DBH); Medium (10 – 20 cm DBH); Large (21 – 30 cm DBH) and Very large (> = 31 cm DBH).

**
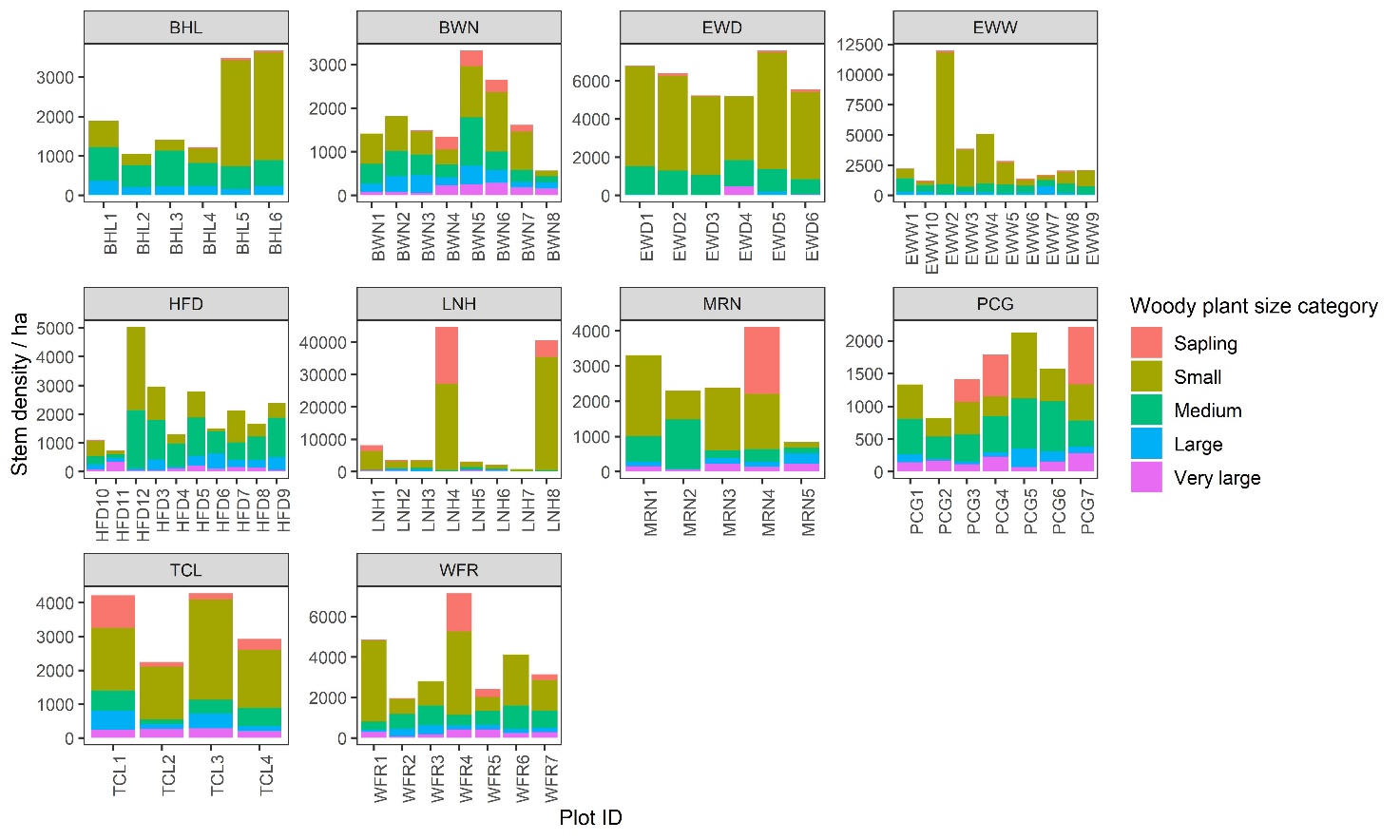
**

**S4:** Average percentage cover of bramble across the ten woodland sites. Error bars represent the standard error of the means.


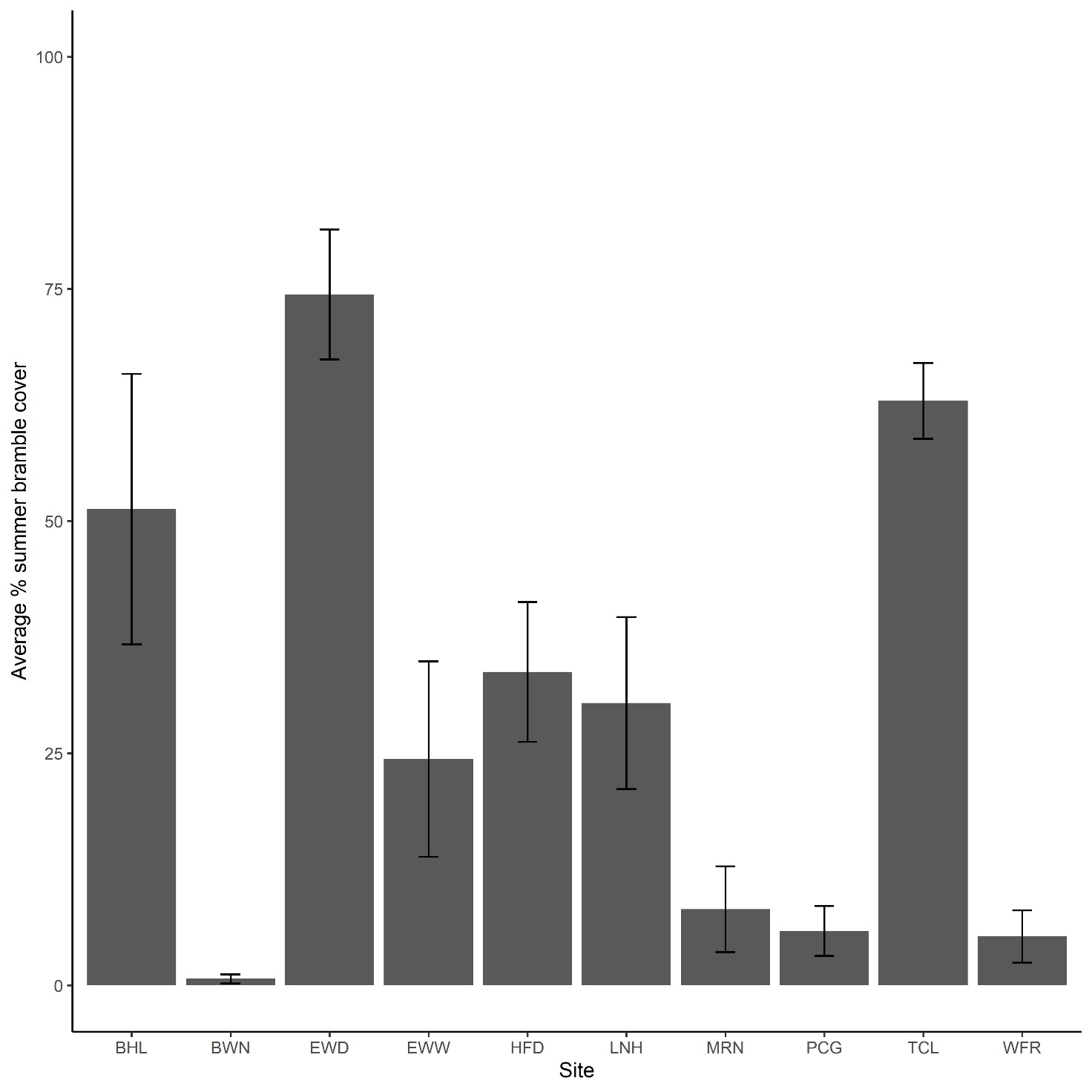


**S5**: Predicted values from a single factor linear mixed model showing that logged density of small stems (y) was significantly negatively related to the average shade intensity from mature trees (x). The error around the line represents the 95% confidence intervals of the predicted values from the model.


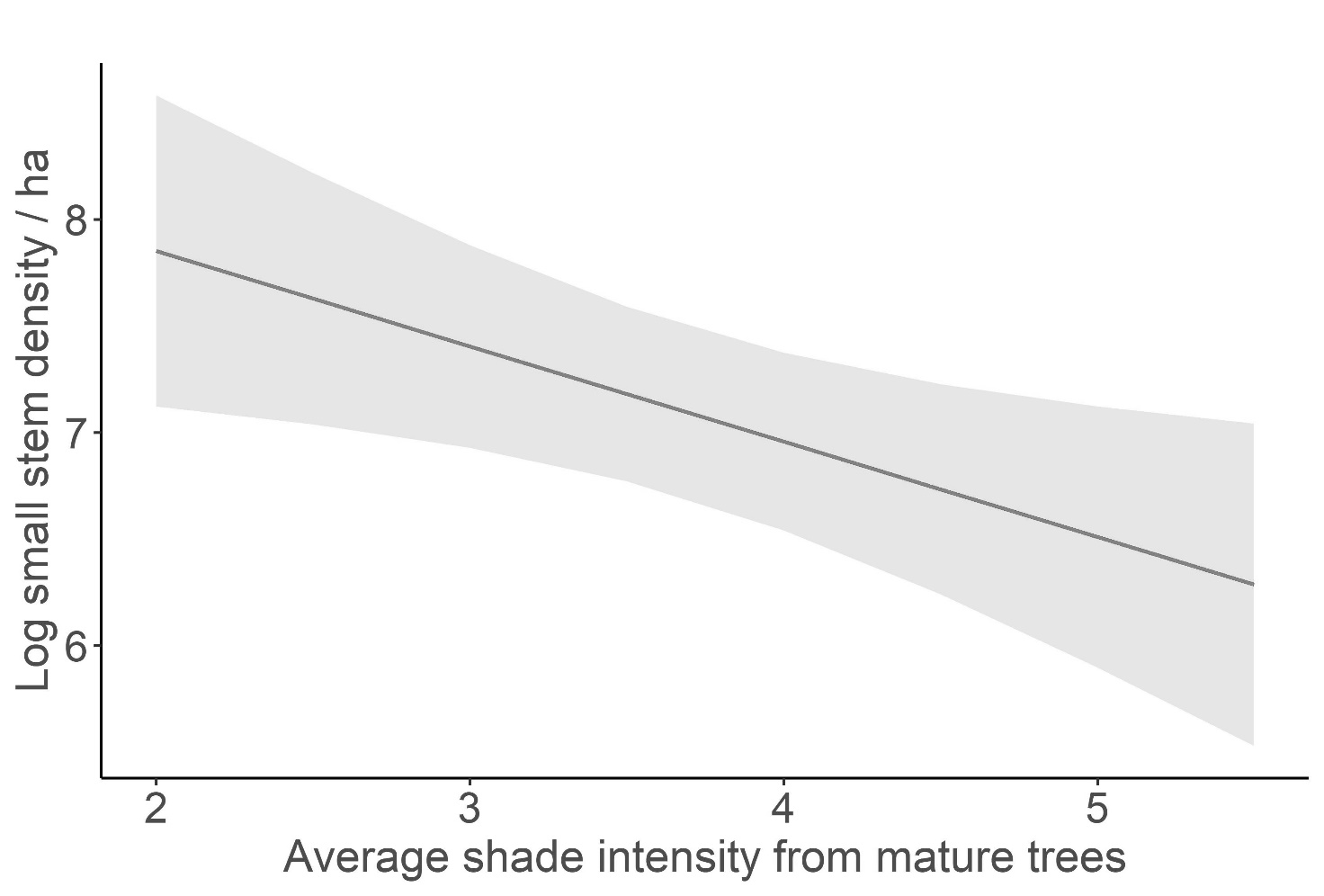


**S6:** Average resting height of survey quadrats during summer bramble cover surveys across the ten study sites. This provides an indication of the height of the field layer vegetation including bramble. Caution is advised when interpreting these data however, for two reasons: 1) there was an effect of the quadrat squashing down vegetation, and 2) it is likely there was surveyor bias of avoiding areas where the field vegetation was so dense that safe access was not possible. For these reasons, these data are likely to underestimate the height of the field layer vegetation, including bramble. The error bars represent the standard error of the means.


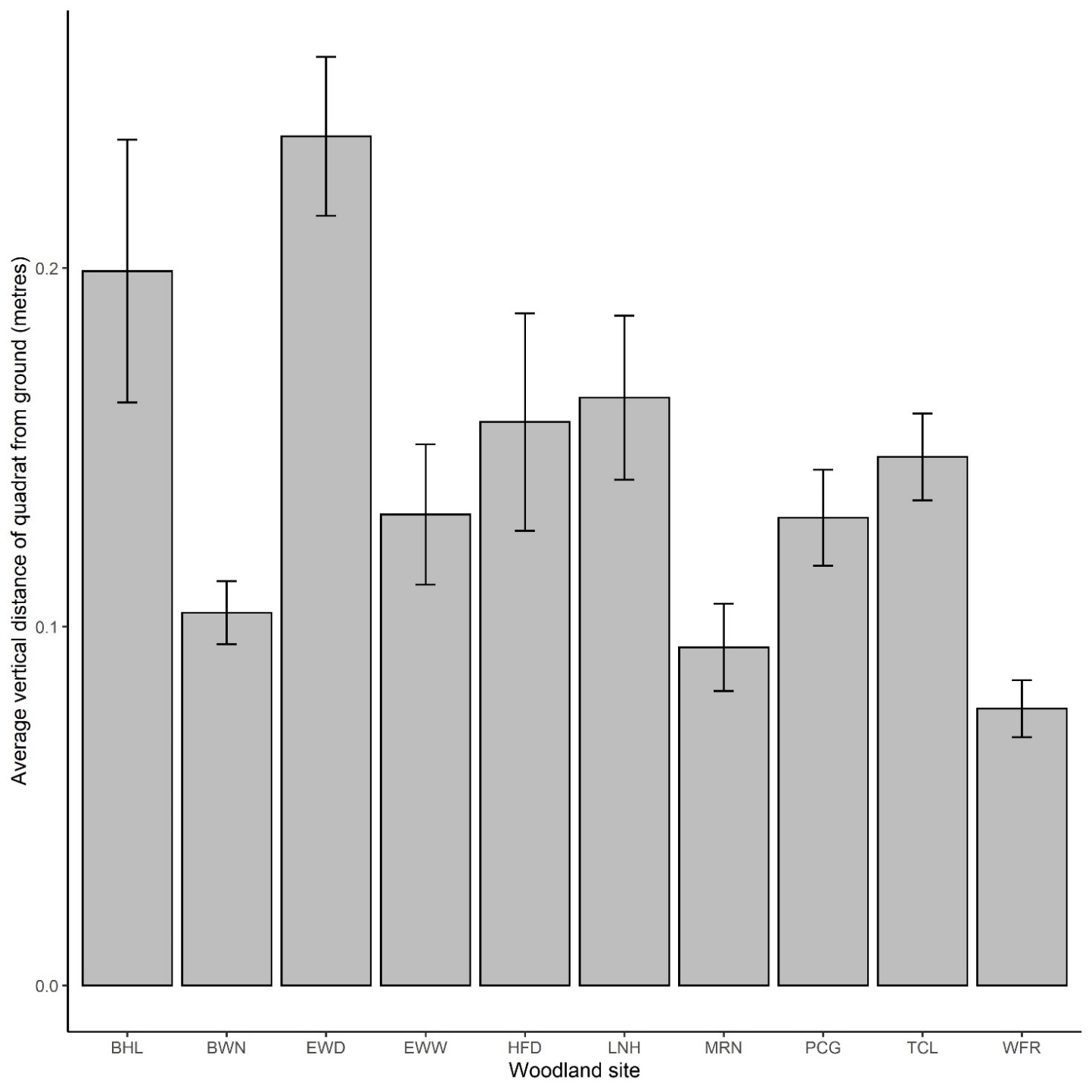


**S7:** Understory Leaf Area Index (LAI) of the ten study sites. LAI measurements were derived from Leaf Area Density (LAD) profiles at 0.75 – 1.5 metres above the ground. Each site mean was taken from between four and ten study plots. The error bars represent the standard error of the means.

**
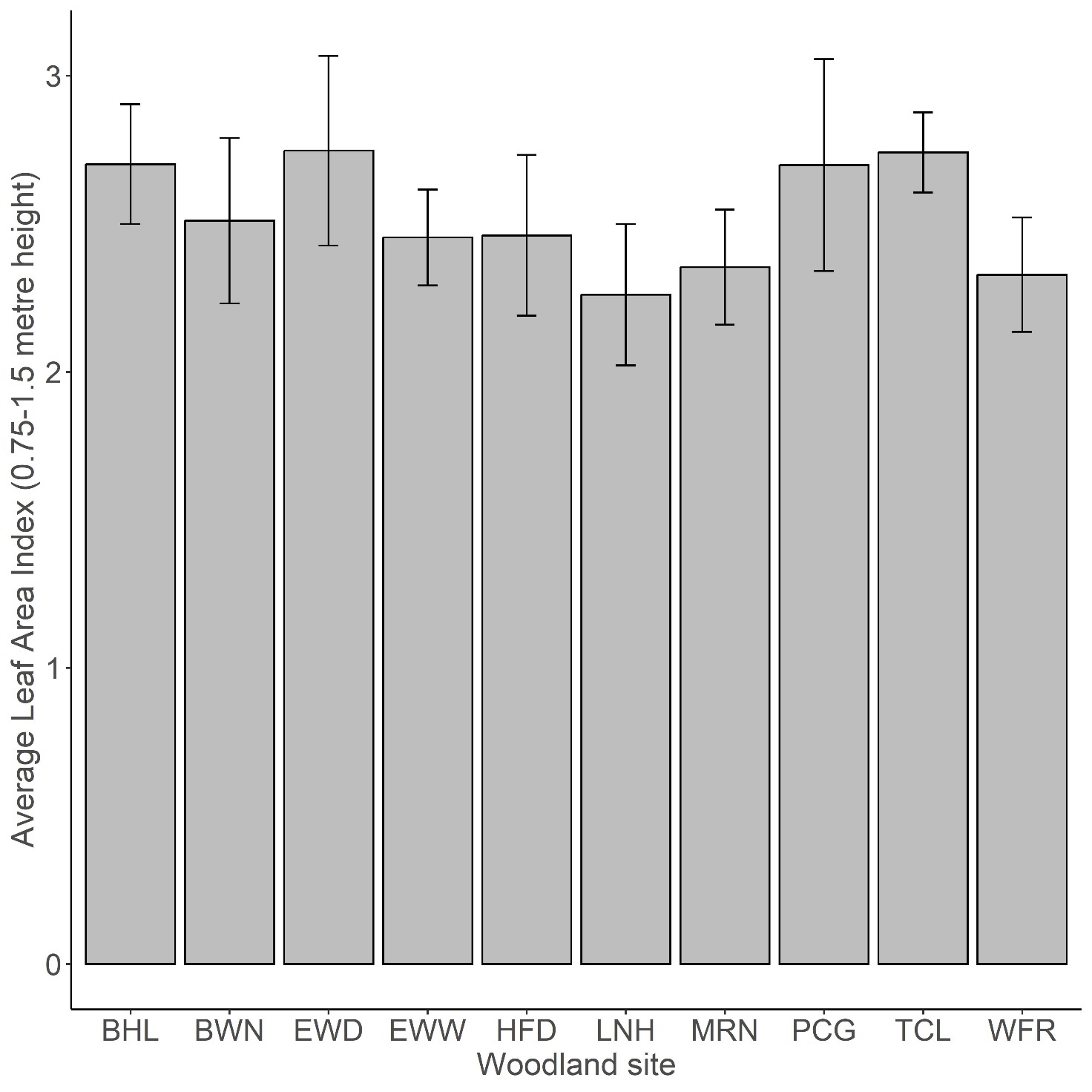
**
